# Butterfly blues and greens caused by subtractive colour mixing of carotenoids and bile pigments

**DOI:** 10.1007/s00359-023-01656-4

**Published:** 2023-07-12

**Authors:** Doekele G. Stavenga

**Affiliations:** Groningen Institute for Evolutionary Life Sciences, University of Groningen, Nijenborgh 7, 9747AG Groningen, The Netherlands

**Keywords:** Reflectance spectrum, Pterobilin, Sarpedobilin, Camouflage, Telesiphepigment

## Abstract

Butterflies often have conspicuously patterned wings, due to pigmentary and/or structurally wing scales that cover the wing membrane. The wing membrane of several butterfly species is also pigmentary coloured, notably by the bile pigments pterobilin, pharcobilin and sarpedobilin. The absorption spectra of the bilins have bands in the ultraviolet and red wavelength range, resulting in blue-cyan colours. Here, a survey of papilionoid and nymphalid butterflies reveals that several species with wings containing bile pigments combine them with carotenoids and other short-wavelength absorbing pigments, e.g., papiliochrome II, ommochromes and flavonoids, which creates green-coloured patterns. Various uncharacterized, long-wavelength absorbing wing pigments were encountered, particularly in heliconiines. The wings thus exhibit quite variable reflectance spectra, extending the enormous pigmentary and structural colouration richness of butterflies.

## Introduction

Butterflies display various colourful wing patterns, which function for display and/or camouflage. Commonly, the wing colours are created by a lattice of scales that cover the wing membrane as shingles on a roof, but sometimes the wing membrane itself is coloured (Rothschild and Mummery 1985; Nijhout 1991; Kronforst et al. 2012). The wing colouration can have a pigmentary and/or structural origin, and in each species, this is spatially organized in characteristic patterns. To adequately understand the function and evolution of the colouration of butterfly wings, knowledge of the specific pigment types together with the wing and scale anatomy is essential.

Pigmentary colouration is due to wavelength-selective absorbing substances. Well-documented pigments in butterfly wing scales are the papiliochromes in papilionids (Umebachi 1985; Wilts et al. 2012a), ommochromes in nymphalids (Nijhout 1997; Reed et al. 2008), and pterins in pierids (Wijnen et al. 2007). The absorption spectra of these pigments can be restricted to the ultraviolet wavelength range, then creating a white colour, but extension into the blue wavelength range causes yellow or orange colours, and with an even broader absorption band red colours result, as occurs in various pierids (Wijnen et al. 2007; Wilts et al. 2017a).

Blue and green colours in insects are usually due to structural colouration mechanisms. For instance, the striking blue structural colour displayed by the iconic *Morpho* butterflies is created by multilayered nanostructures in their wing scales (Ghiradella 1998; Kinoshita et al. 2002; Vukusic and Sambles 2003; Yoshioka and Kinoshita 2006; Kinoshita 2008; Giraldo and Stavenga 2016). The green wings of the lycaenids *Callophrys rubi* and *Thecla opisena* are due to gyroid structured scales (Michielsen et al. 2010; Wilts et al. 2017b). Purple can be made by combining a red pigmentation with a blue structural colour, as in the Queen purple tip, *Colotis regina*, which is a case of additive colour mixing (Giraldo et al. 2008).

Brown to black scales, caused by melanin pigment, are universally present in members of all butterfly families. Melanin sometimes also exists in the wing membrane (Yoshioka and Kinoshita 2006), but most butterfly wing membranes are more or less transparent. Nevertheless, several butterfly species have wing membranes that are coloured by various pigments, a theme that has received little attention in the last decades, especially as those colours are blue to green.

In 1898, Baer reported that the swallowtails *Graphium antheus*, *Papilio phorcas* and *G. agamemnon*, as well as the nymphalids *Philaethria dido* and *Parantica cleona*, have green-pigmented wing membranes (Baer 1898). Later analyses of the green wings and skin of Orthoptera showed that the green colour was due to the combined effect of blue bile pigment and yellow carotenoid, an example of subtractive colour mixing (Okay 1945, 1951), similar as in the green hemolymph and skin of the larvae of numerous insect species (Junge 1941; Hackman 1952; Willig 1969).

The green colouration clearly has a function in camouflage and crypsis (Jin and Fujiwara 2017). Indeed, the bile pigments (bilins) together with carotenoids notably colour the epidermis of lepidopteran larvae as well as pupae (Junge 1941; Ohtaki and Ohnishi 1967; Shirataki et al. 2010; Jin et al. 2019), but in the large majority of adult butterflies they are replaced by various other pigments, the papiliochromes, ommochromes, pterins and melanins, mentioned above. Consequently, the function of bilins for the colouration of adult butterflies has received little attention, and the same holds for the carotenoids, even though various carotenoids were shown to exist in the wing membranes of numerous papilionids, nymphalids and pierids (Feltwell and Rothschild 1974; Allyn et al. 1982; Rothschild and Mummery 1985). The aim of this paper therefore is to re-examine the butterfly blues and greens.

Choussy extensively assayed the wings of papilionid, nymphalid, and pierid butterfly species, and also several nocturnal lepidopteran species, for the occurrence of bilins (Bois-Choussy 1977). She thus demonstrated the ubiquitous presence of the bile pigment pterobilin. It belongs to the tetrapyrroles, the class of pigments containing biliverdin, the green pigment of locusts (Mahamat et al. 1997), as well as chlorophyll and phytochrome, universal pigments of plants (Bryant et al. 2020). Together with Barbier, Choussy studied the complex chemistry of various butterfly bile pigments and their precursors by focusing on the papilionids *P. phorcas* and *Graphium sarpedon* (Choussy et al. 1973; Choussy and Barbier 1973; Bois-Choussy 1977; Barbier 1986). Their specific pigment types, phorcabilin and sarpedobilin, were demonstrated to be formed by cyclization of the basic pterobilin (Bois-Choussy and Barbier 1983).

The absorption spectra of the pigments are crucial for a quantitative understanding of how the bile and carotenoid pigments are giving colour to the Lepidoptera. The spectra of the various bilins derived from in situ spectrophotometry appeared to depend on the species, but the spectra reported by Bois-Choussy (1977) were somewhat confusing (Stavenga 2023). The latter may be due to the condition of the pigments, because extensive studies on larvae demonstrated that there both bilins and carotenoids are bound to proteins, which can substantially affect the pigments’ absorption spectrum (Huber et al. 1987; Scheer and Kayser 1988; Iturraspe et al. 1989; Jin and Fujiwara 2017).

Investigations of the anatomical location of the blue/green pigments in the wings of a number of *Graphium* species showed that the pigments exist in the wing membrane and especially in the wing veins, where the fluid ‘haemolymph is bright green when it oozes from a severed vein; the presence of carotenoids adds to the particular yellowish-green tone of this fluid’ (Allyn et al. 1982). The latter authors stated that the carotenoids are scarce in *G. sarpedon* (Allyn et al. 1982), but more recent experiments demonstrated that the wing membrane areas with a distinctly green colour contained a considerable amount of the carotenoid lutein (Stavenga et al. 2010). In subsequent optical studies on the colouration of butterfly wings, pigments with an absorption band in the long-wavelength range, reminiscent of sarpedobilin, were encountered (Stavenga et al. 2010, 2020; Wilts et al. 2017c). The present study highlights the occurrence of bilins and carotenoids (as well as of other short-wavelength absorbing pigments) in several butterfly species and their possibly significant role for the colouration of adult butterfly wings. In combination with other wing pigments, they realize the striking beauty of butterfly wing patterns.

## Materials and methods

### Specimens

Mounted butterfly specimens were obtained from various commercial sources: World Wide Butterflies Ltd, thebugmaniac.com, Insect-Sale.com, demuseumwinkel.com. Pupae were purchased from Papiliorama (Havelte, Netherlands).

### Spectrophotometry

Reflectance spectra were measured with a bifurcated probe connected to a halogen/deuterium light source and an Avantes AvaSpec-2048-2 CCD detector array spectrometer (Avantes, Apeldoorn, Netherlands). The measurement area was of the order of 1 mm^2^. The measured signal was divided by the signal obtained from a white diffuse standard (Avantes WS-2), which thus served as a reference. To determine the absorption spectra of the wing pigments, transmittance spectra of small areas (10 × 10 µm^2^) of wing pieces were measured with a microspectrophotometer (MSP), consisting of a Leitz Ortholux microscope with a LUCPlanFL N 20x/0.45 objective (Olympus, Tokyo, Japan) and the Avantes spectrometer. To strongly reduce the reflection and scattering by the inhomogeneities of the wing scales and membrane, the wing pieces were positioned at a microscope slide, embedded in immersion oil (refractive index 1.515), and then covered by a cover slip. The reference was an area adjacent to the embedded wing piece. The transmittance spectra, *T*, were converted into absorbance spectra via *A* = −log10(*T*). As the shapes of reflectance and transmittance spectra are very similar, the reflectance spectra, *R*, were similarly converted into absorbance spectra via *A* = −log10(*R*). The latter resulting spectra should not be confused with spectra obtained from transmittance measurements.

## Results

### Carotenoids and bile pigments in the wings of Papilionidae

**Fig. 1:**
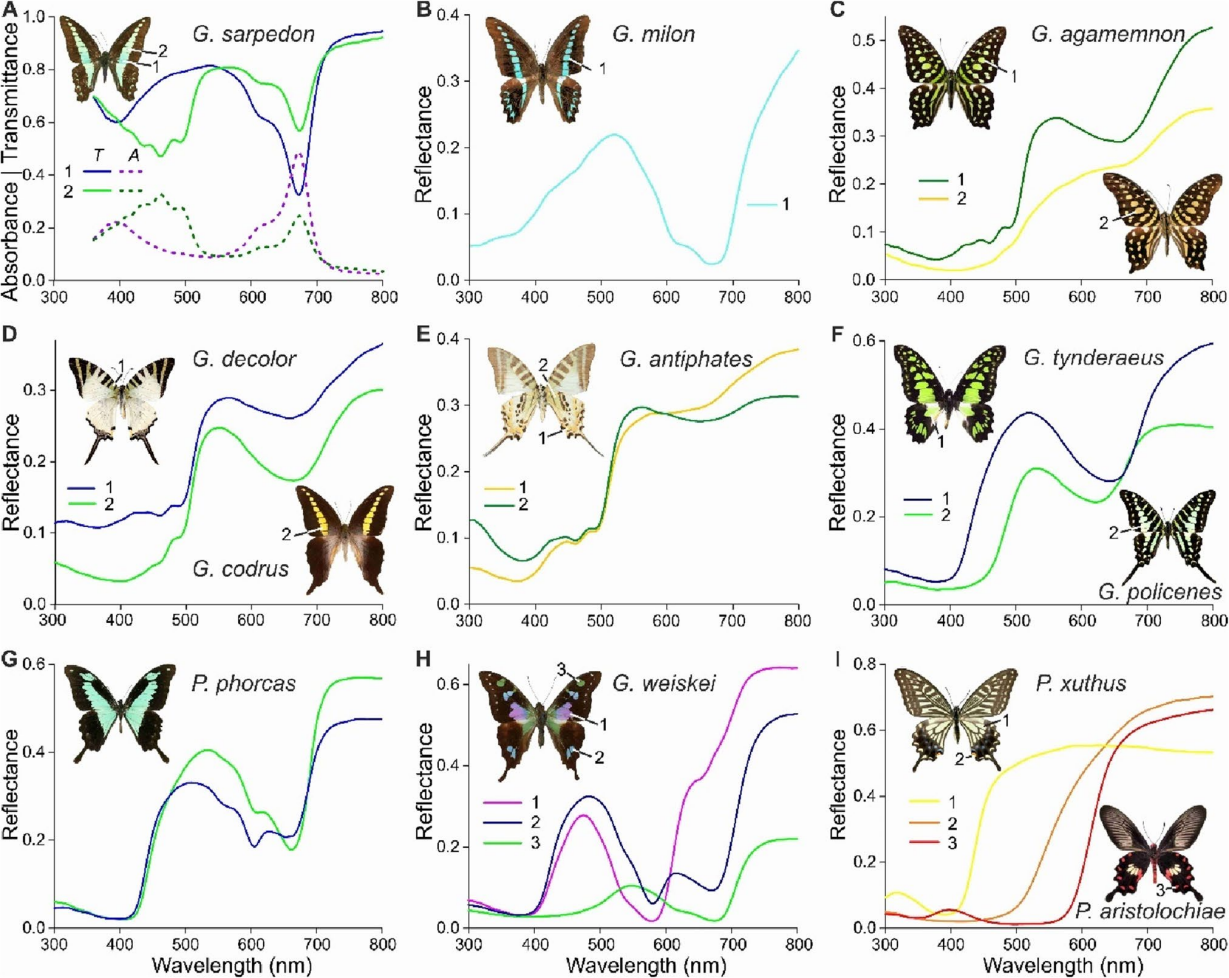
Spectral characteristics of some papilionids. **A** Common bluebottle, *Graphium sarpedon*. *T*: transmittance spectra of blue (1) and green (2) wing areas. *A*: absorbance spectra calculated from *T*. **B** *Graphium milon*. Reflectance spectrum of blue wing area (1). **C** Tailed jay, *Graphium agamemnon*. Reflectance spectra measured from two different specimens. **D** Swordtails *Graphium decolor* and *Graphium codrus*. Reflectance spectra of green-yellow wing areas. **E** Five-bar swordtail, *Graphium antiphates*. Reflectance spectra of two slightly different yellow wing areas. **F** Green kite and Small striped swordtail, *Graphium tyndaraeus* and *Graphium policenes*. Reflectance spectra of green wing areas. **G** Apple-green swallowtail, *Papilio phorcas*. Reflectance spectra of cyan-green wing areas. **H** Purple spotted swallowtail, *Graphium weiskei*. Reflectance spectra of purple (1), blue (2), and green (3) wing areas. **I** *Papilio xuthus* and *Pachliopta aristolochiae*. Reflectance spectra of yellow, orange and red wing areas

*Graphium sarpedon* is a prominent papilionid species with bile pigment and carotenoid in the wing membrane (Stavenga et al. 2010). Figure 1A presents transmittance spectra of a cyan and green coloured wing area, both showing a distinct valley at 672 nm. The green wing area has a reduced transmittance in the blue wavelength range, characteristic for the carotenoid lutein (Jouni and Wells 1996). The transmittance (*T*) of the cyan wing converted into absorbance (*A*) yields the spectrum for the bile pigment sarpedobilin (Fig. 1A, #1), and the absorbance spectrum of the green wing area shows its pronounced lutein content (Fig. 1A, #2). The *G. sarpedon* spectra enable understanding of the colouration of other papilionid butterflies, for instance, the wing pattern of *G. milon*. *G. milon* was long treated as a subspecies of *G. sarpedon*, but is now considered to be a subspecies of *G. anthedon* (Vane-Wright and de Jong 2003). Its pronounced, blue-coloured wing band resembles that of *G. sarpedon*, but the colouration is uniform (Fig. 1B). The coloured wing band has a strongly reduced reflectance between 600 and 700 nm, which can be immediately understood with the sarpedobilin spectrum of Fig. 1A. The reflectance in the blue wavelength range indicates only a minor presence of carotenoid.

In comparison, the wing pattern of *G. agamemnon* is more elaborate and the colour is greenish to yellow, depending on the specimen (Fig. 1C). A green colour results from a mixture of carotenoid (probably lutein) and bile pigment (Fig. 1C, #1), as in *G. sarpedon* and *G. milon* (Fig. 1A, B), but the wings of *G. agamemnon* contain pterobilin (Choussy and Barbier 1973; Bois-Choussy 1977). The absorption spectrum of sarpedobilin peaks at 672 nm and has a shoulder at ~ 610 nm (Fig. 1A), so that the reflectance spectrum of the blue wings of *G. milon* has a valley around 670 nm with a slight twist at 610 nm (Fig. 1B). Pterobilin has a monophasic absorption spectrum in the red wavelength range (Wieland and Tartter 1940; Bois-Choussy and Barbier 1983), so that the reflectance valley in the red wavelength range of green-winged *G. agamemnon* is smooth, with a minimum at 660 nm. The reflectance spectra of green-yellow *G. agamemnon* wings (Fig. 1C, #2) show a minor pterobilin content, and the lack of oscillations in the blue wavelength range indicate the presence of a pigment that is different from the carotenoids. The obvious candidate is papiliochrome II, the pigment commonly encountered in papilionid wings (e.g., in *Papilio xuthus*, Fig. 1I, #1).

Reflectance spectra similar to those of *G. agamemnon* are obtained from the wings of *G. decolor* and *G. codrus*, showing variable mixtures of carotenoid and pterobilin (Fig. 1D). The wings of *G. antiphates*, *G. tynderaeus* and *G. policenes* were reported to contain pterobilin (Bois-Choussy 1977), which is confirmed by their reflectance spectra, which have minima at 660–665 nm (Fig. 1E, F). However, *G. tynderaeus* and *G. policenes* apparently lack carotenoids (Fig. 1F), and the low UV reflectance suggests a substantial concentration of (presumably) papiliochrome II.

The pigments thus appear to be rather variable, dependent on the species and the specimen. This is also demonstrated in the related papilionid species *P. phorcas* (Fig. 1G) and *G. weiskei* (Fig, 1H). The reflectance spectra of *P. phorcas* wings show a very low reflectance in the ultraviolet, indicating again a substantial amount of papiliochrome, and there are two reflectance dips, at 604 and 663 nm (Fig. 1G). The latter are due to two bilins, phorcabilin and sarpedobilin, respectively (Bois-Choussy 1977). The case of *G. weiskei* is even much more complex. The green wing patches are created by the combined action of a slightly modified sarpedobilin with absorption peak wavelength 676 nm and a UV-violet absorbing pigment, presumably papiliochrome (Fig. 1H, #3). The reflectance spectrum of the blue wing areas has minima at 676 and 580 nm, due to about equal amounts of sarpedobilin and another pigment, provisionally called weiskeipigment (Fig. 1H, #2). The latter pigment causes the purple-coloured patches of *G. weiskei*, which contain only a trace of sarpedobilin (Stavenga 2023).

Figure 1I is included here, to show the wing reflectance spectra resulting from papiliochrome II and related kynurenine-based pigments of papilionids (Wilts et al. 2012a; Nishikawa et al. 2013). These pigments are all optical longpass filters, quite different from the bilins that act as blue-green bandpass filters.

### Carotenoids and bile pigments in the wings of Nymphalidae

**Fig. 2:**
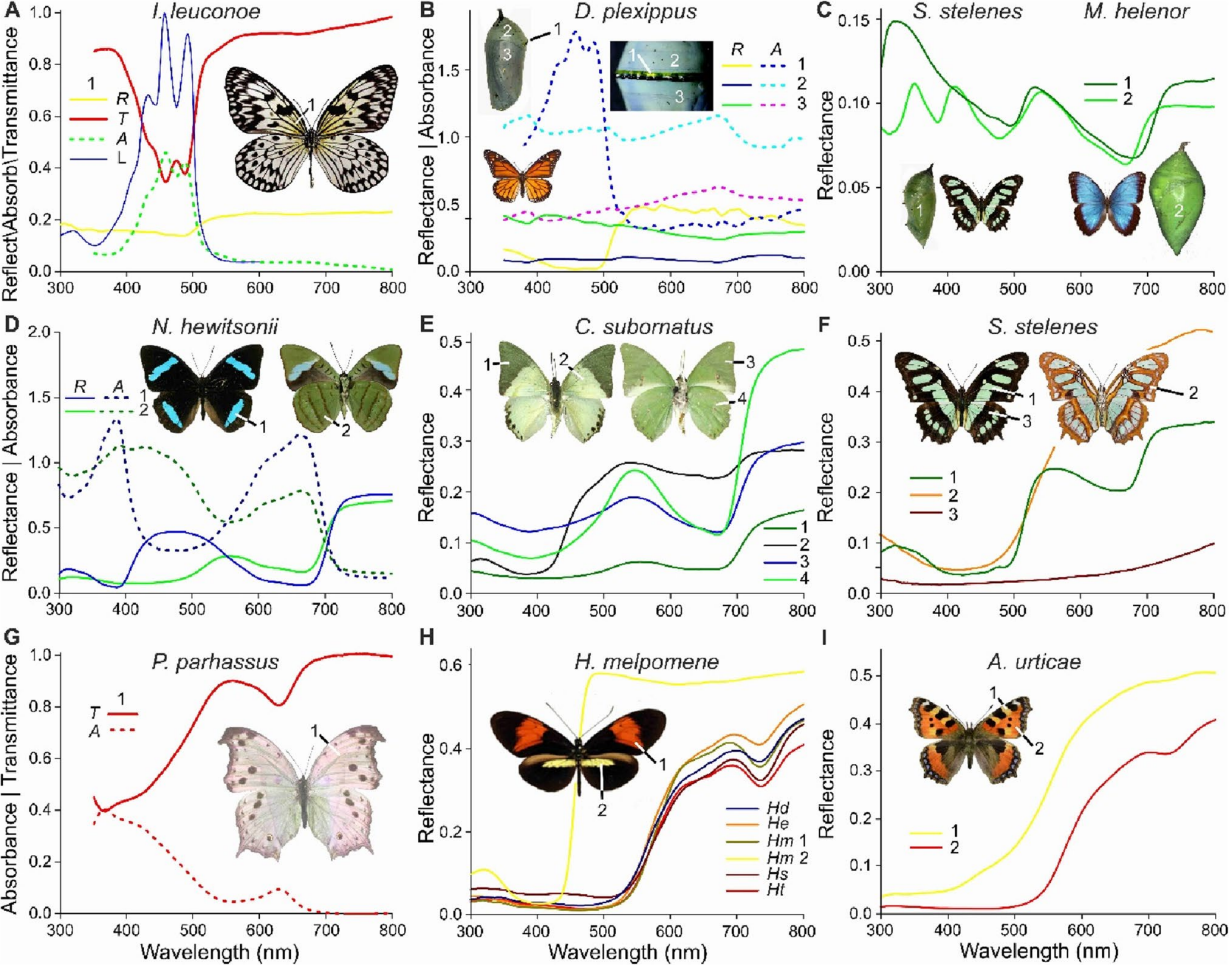
Spectral characteristics of some nymphalids. **A** Paper kite butterfly, *Idea leuconoe*. *R*: reflectance spectrum of yellow central forewing area (1). *T*: transmittance spectrum of a wing piece embedded in immersion oil. *A*: absorbance spectrum calculated from *T*. *L*: absorption spectrum of lutein. **B** Monarch, *Danaus plexippus*. *R*: reflectance spectra of monarch pupa from location 1 (a yellow-reflective spot in the diadem, running along the edge of the anterior keel), location 2 (greenish top area), and location 3 (more bluish lower area). *A*: absorbance calculated from *R*. **C** Malachite, *Siproeta stelenes* and Common blue morpho, *Morpho helenor*. Reflectance spectra of green pupae. **D** Hewitson’s olivewing (*Nessaea hewitsonii* (inset left: dorsal, right ventral). *R*: reflectance spectra of dorsal blue band (1) and ventral green area (2). *A*: absorbance calculated from *R*. **E** Ornate green charaxes, *Charaxes subornatus* (inset left: dorsal, right ventral). Reflectance spectra of areas 1–4. **F** Malachite, *Siproeta stelenes* (inset left: dorsal, right ventral). **G** Common mother-of-pearl, *Protogoniomorpha parhassus*. *T*: transmittance spectrum of a wing piece embedded in immersion oil measured with a microspectrophotometer. *A*: absorbance spectrum calculated from *T*. **H** Heliconiines, *Heliconius doris* (*Hd*), *H. erato* (*He*), *H. melpomene* (*Hm*), *H. sara* (*Hs*), and *H. telesiphe* (*Ht*). Reflectance spectra of red wing areas for all species; for *H. melpomene* spectra of both red (1) and yellow (2) wing areas. **I** Small tortoiseshell *Aglais urticae*. Reflectance spectra of yellow (1) and red (2) wing areas

The wings of the nymphalid *Idea leucone* display an irregular checkerboard of black, melanised spots, but the proximal wing areas feature a yellow colour (Fig. 2A). Whereas the wing reflectance (*R*) spectrum only reveals a rather shallow suppression, the transmittance (*T*) spectrum of a wing piece in immersion oil is again a clear signature of the carotenoid lutein. Converted into the absorbance (*A*) spectrum, it closely corresponds with the absorption spectrum of lutein. Lutein is also the prominent carotenoid in the pupae of the monarch, *Danaus plexippus*, especially in the ‘golden diadem and small gold flecks’, where the yellow reflections (Fig. 2B, R #1) are enhanced by multilayer reflectors (Rothschild et al. 1978). Calculating the absorbance as *A* = −log10(*R*) yields a lutein-like spectrum (Fig. 2B, A #1). The pupae reflections on both sides of the diadem are blue-greenish, with depressions in the red wavelength range at ~ 670 nm (Fig. 2B; *R* #2, 3). The reflectance spectra converted into absorbance spectra show peaks that indicate the presence of pterobilin or a closely related bile pigment (Fig. 2B, A #2, 3).

Pupae of *Siproeta stelenes* (Nymphalinae) and *Morpho helenor* (Satyrinae) have similar colour characteristics, with a distinctly low reflectance at ~ 670 nm, revealing prominent bile pigment in the haemolymph (Fig. 2C). The pigments responsible for the reflectance in the short-wavelength range are unclear, but the minor oscillations in the reflectance spectrum of the *S. stelenes* pupa between 400 and 500 nm may indicate the participation of carotenoid. Similar minor oscillations can be seen in the absorbance spectra calculated from the reflectance spectra of green ventral wings of *Nessaea hewitsonii* (Biblinidae; Fig. 2D, #2). More importantly, the dorsal wings are marked by deep-blue bands, but neither their reflectance nor absorbance spectra have in the red wavelength range a simple rounded shape, expected for pterobilin. Pterobilin is present in the wings of a member of the same genus, *Nessaea obrinus*, which has dorsal forewings with similar blue bands (Choussy and Barbier 1973; Bois-Choussy 1977). Whether *N. hewitsonii* wings contain a modified pterobilin or a mixture of bilins needs further study.

The wings of *Charaxes subornatus* as well its relative *Charaxes eupale* (Charaxinae) contain pterobilin (Bois-Choussy 1977). The wing colour is overall greenish, with the distal part of the dorsal forewing being dark green, due to a lattice of alternating green and black scales (Fig. 2E, #1). The remaining part of the dorsal wings is white, due to scales with a low UV reflectance (presumably due to kynurenine; Fig. 2E, #2), and the ventral wings are light-green coloured (Fig. 2E, #3, 4). The reflectance spectra of the green wing areas show a valley in the red wavelength range, in agreement with the reported occurrence of pterobilin.

The green wing part of *S. stelenes* (Nymphalinae) also contains pterobilin (Bois-Choussy 1977), and the reflectance spectrum shows the corresponding valley in the red (Fig. 1F, #1). The orange-coloured framework of the ventral wings will be caused by an ommochrome-type pigment (Fig. 2F, #2; see Fig. 2I, #1). The black framework of the dorsal wings is clearly due to strongly melanic scales, as indicated by the low reflectance that gradually increases with increasing wavelength (Fig. 2F, #3). The related *Protogoniomorpha parhassus* (also Nymphalinae) has an overall pink colour, thanks to scales acting as thin film reflectors, but the transmittance spectrum shows that the wings harbour a short-wavelength absorbing pigment (probably ommochrome) and a quite special pigment with an absorption band in the red wavelength range, peaking at 630 nm (Fig. 2G), distinct from bile pigment peaking at ~ 670 nm (Fig. 2E #4, F #1; Stavenga 2021).

An even more deviant case is that of heliconiines that have wings featuring red scales (Fig. 2H). Their reflectance spectra reveal a broad-band absorber in the visible wavelength range, most likely an ommochrome, together with another pigment that has a distinct absorption band peaking at 735 nm (Fig. 2H, #1; Wilts et al. 2017c). The yellow wing areas lack that pigment (Fig. 2H, #2). Comparable phenomena are seen in the reflectance spectra of nymphalines, e.g., *Aglais urticae* (Fig. 2I, #1, 2) and *Vanessa atalanta* (Stavenga et al. 2014). Alike the heliconiines, the unknown pigment is lacking in the yellow wing areas of the nymphalines (Fig. 2I, #1), but the shallow depression at 735 nm in the spectrum of the red wing area of the nymphaline (Fig. 2I, #2) indicates a small concentration of the pigment (see also Fig. 1 of Stavenga et al. 2014).

## Discussion

### Bilins and the effect of protein binding

**Fig. 3:**
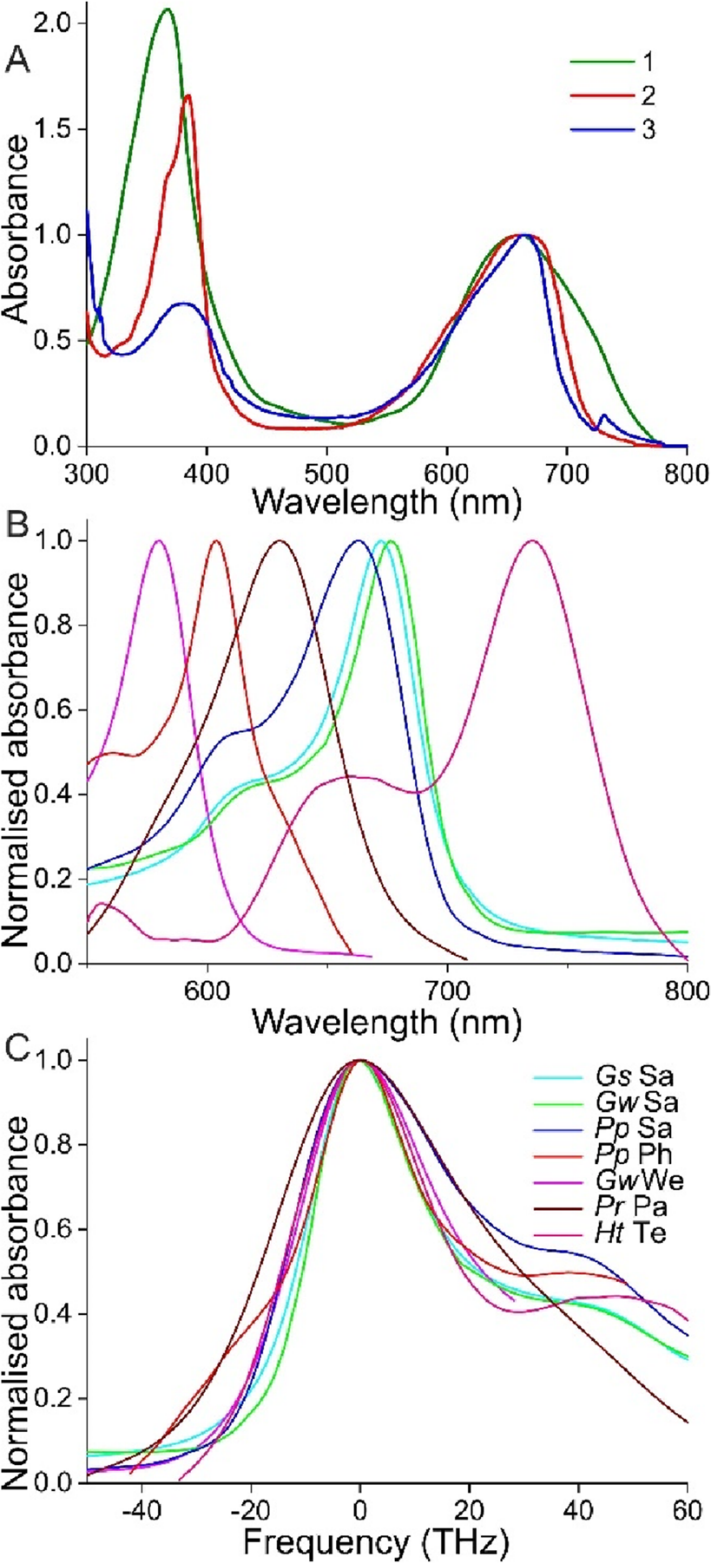
Spectral characteristics of the long-wavelength absorbance bands of butterfly bile-like pigments. **A** Absorption spectra of pterobilin dimethylesther in metanol (#1; derived from Fig. 2 of Bois-Choussy and Barbier 1983) and bilin binding protein BBP of *Pieris brassicae* (#2; derived from Huber et al. 1987), together with biliverdin binding protein BP-2 of the noctuid moth *Spodoptera litura* (#3; derived from Yoshiga and Tojo 1995). The spectra are normalized at the long-wavelength peak. **B** Normalised absorbance spectra plotted as a function of wavelength of sarpedobilin (Sa), phorcabilin (Ph), weiskeipigment (We), parhassuspigment (Pa), and telesiphepigment (Te) obtained for *Graphium weiskei* (*Gw*), *G. sarpedon* (*Gs*), *Papilio phorcas* (*Pp*), *Protogoniomorpha parhassus* (*Pr*), and *Heliconius telesiphe* (*Ht*). **C** The spectra plotted as a function of frequency relative to the peak frequency

Pterobilin, the most common bilin, was first isolated from cabbage butterflies, *Pieris brassicae* (Wieland and Tartter 1940). The chemical characteristics have been extensively studied in extracts (Bois-Choussy 1977), but the reported spectral properties of the extracts conflict with the in situ measurements. The isolated pigment absorbs strongly in the ultraviolet and red wavelength ranges (Fig. 3A, #1; from Bois-Choussy and Barbier 1983), thus creating a blue-cyan colour. However, the bilins are in situ bound to proteins, which can modify the absorption spectrum (Huber et al. 1987; Scheer and Kayser 1988; Iturraspe et al. 1989; Jin and Fujiwara 2017). In the case of pterobilin, this may be of minor importance, but in the related pharcobilin and sarpedobilin, which are derived from pterobilin by cyclisation (Bois-Choussy 1977), protein binding can determine the conformation (Iturraspe et al. 1989). This not only can affect the absorption ratio of the UV and red bands (Fig. 3A, #1, 2), but the spectrum can also considerably depend on the specific bilin-binding protein. For instance, from larvae of the saturniid silkworm *Rhodinia fugax*, two different blue biliproteins, BP-I and BP-II were isolated, which bind phorcabilin and pterobilin with peak absorbance in the red wavelength range at 669 and 663 nm, respectively (Saito 1998). Spectrophotometry on *P. phorcas* wings, which contain predominantly phorcabilin, but also sarpedobilin (Bois-Choussy 1977), showed that the absorbance peak wavelengths of pharcobilin and sarpedobilin are 604 and 663 nm (Stavenga 2023). This suggests that the bilin-binding proteins of *P. phorcas* distinctly differ from those of *R. fugax*. Also, from larvae of the noctuid moth *Spodoptera litura*, four different biliverdin-binding proteins (BP1-4), closely related to bilin-binding protein, were purified; their joint absorbance spectrum is broader than that of the isolated BP-2 (Fig. 3A, #3; Yoshiga and Tojo 1995). Further study will elucidate whether the measured spectra are the result of only one pigment state or that a combination of pigment conformations causes the shape of the spectra.

The spectral measurements of the papilionids and nymphalids of Figs. 1 and 2 demonstrate the presence of various long-wavelength absorbing pigments, reminiscent of the bilins (Fig. 3B, C). Previously, the sarpedobilins (Sa) of *G. sarpedon* (*Gs*), *G. weiskei* (*Gw*) and *P. phorcas* (*Pp*) were estimated to have absorbance bands with peak wavelengths 672, 676, and 663 nm, respectively. For *P. phorcas*, the peak wavelength of the pharcobilin (Ph) absorbance band is estimated to be 604 nm, and that of the weiskeipigment (We) is 580 nm (Stavenga 2023). The long-wavelength absorbance bands of *Protogoniomorpha parhassus* (*Pr*) and *Heliconius telesiphe* (*Ht*) are due to unkown pigments, here called parhassuspigment (Pa) and telesiphepigment (Te), and their peak wavelengths are 630 and 735 nm, respectively. The telesiphepigment exists in the red wing scales of all studied heliconiines (Fig. 2H). The absorbance bands of the pigments in Fig. 3B have a similar shape, which is apparent from plotting the bands on a frequency scale relative to the peak frequency (Fig. 3C). Whether the weiskeipigment, parhassuspigment and telesiphepigment are modified bilins has to be further investigated.

### Carotenoids and short-wavelength filtering of bilins

*Idea leuconoe* appears to be a rare case where only carotenoid is used, resulting in yellow-coloured wings (Fig. 2A), but the blue-absorbing carotenoid lutein in *G. sarpedon*, combined with the red-absorbing bilin sarpedobilin, colours the wing green (Fig. 1A). In several butterfly species with green-coloured wings, the bilin is pterobilin, as follows from the smooth spectral bands in the long-wavelength range. In many cases, the short-wavelength range also lacks the characteristic fine structure of carotenoids, which suggests that the acting blue filter then is papiliochrome II or 3-OH-kynurenine (Figs. 1F, G and H and 2D, E, G and H). Similarly, the wings of the danaid *Parantica aspasia* have a yellow patterning resembling that of *Idea leuconoe*, but the (unpublished) reflectance spectrum is smooth. More detailed analyses are required to identify the chemical nature of those wing pigments.

### Long-wavelength vs. short-wavelength filtering

The species where the wing reflectance spectrum has a minimum at ~ 660 nm, *G. agamemnon*, *G. decolor*, *G. codrus* and *G. antiphates* (Fig. 1C–E), presumably have wings with pterobilin. *G. tyndareus* and *G. policenes* slightly deviate, as the reflectance minima are at 640 and 620 nm, which is possibly due to modified or as yet unknown bilins (Fig. 1F). Those bilins with absorbance bands with peak wavelength ≤ 670 nm can function to suppress the wing reflections in the red wavelength range, but this seems not to hold for the 735 nm peaking telesiphepigment, as heliconiine vision is limited to below 700 nm (Belušič et al. 2021; McCulloch et al. 2022). Its function may be to suppress the wing reflections in the ultraviolet wavelength range, because bilins have a considerable UV-absorption band.

This function is also fulfilled by the UV-absorbing flavonoid pigment sequestered by the lycaenid *Polyommatus icarus*, which decreases the UV reflectance of the wings of females, so increasing the attractiveness for mate-searching males (Knüttel and Fiedler 2001; Knüttel 2003). UV-absorbing flavonoids have been amply demonstrated in the wings of several lycaenids as well as papilionids and nymphalids (Ford 1941; Wilson 1986, 1987), but their role in tuning the visual display of the butterflies’ wings awaits further study.

### Green colouration for camouflage

**Fig. 4:**
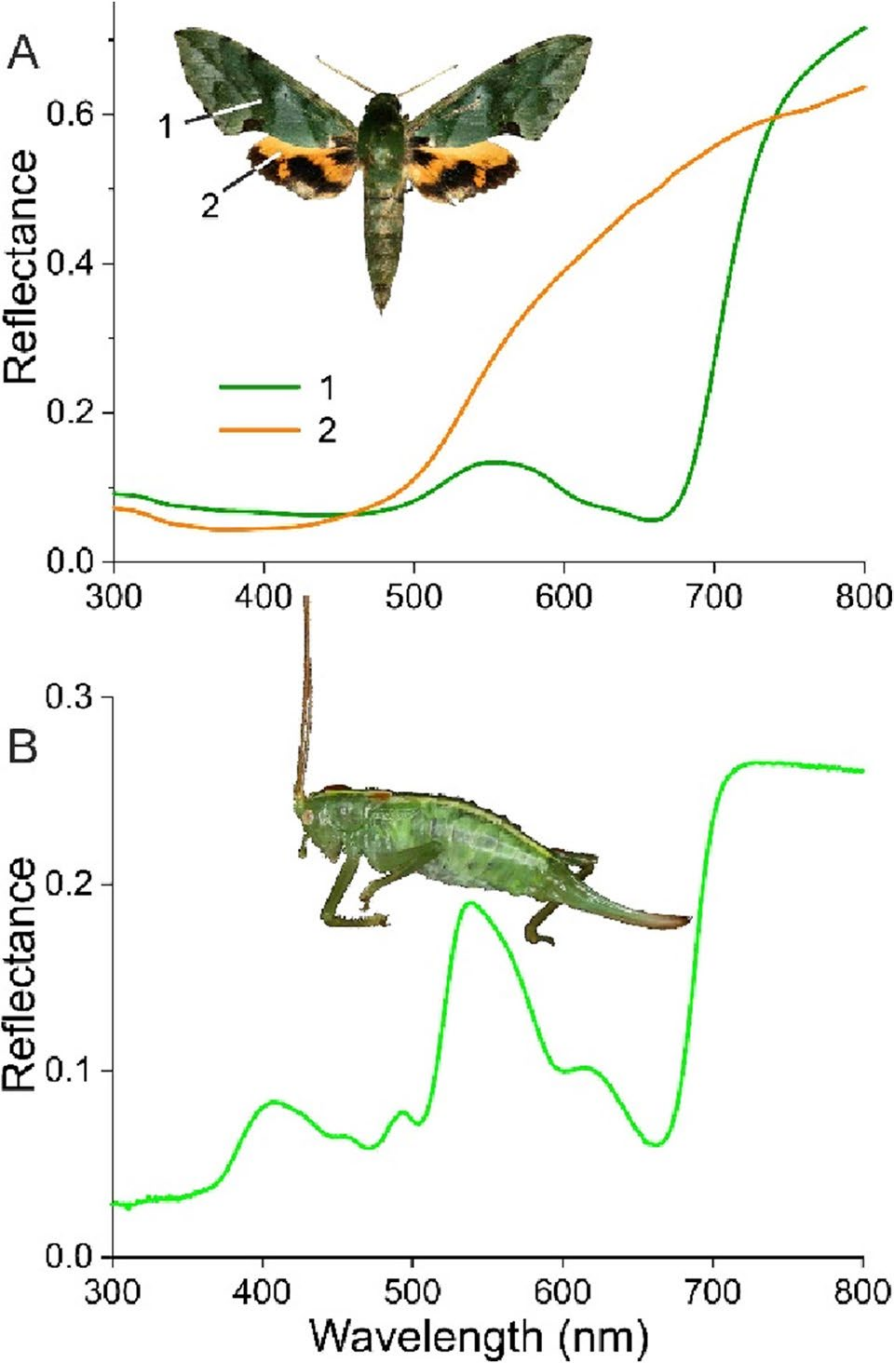
Spectral characteristics of a moth and a bush-cricket. **A** Reflectance spectra of the Verdant hawkmoth, *Euchloron megaera*. **B** Reflectance spectrum of the Oak bush-cricket, *Meconema thalassinum*

The spectral data of Figs. 1 and 2 show that carotenoids and bilins occur in the pupae and wings of several butterfly species, and so cause the butterfly’s green colouration. Green-coloured wings are also encountered in moths, specifically the Geometrinae, eponymously called Emerald moths. They derive their colour from a single pigment, called geoverdin, which is not a bile pigment but possibly a derivative of chlorophyll and thus presumably derived from plant food (Cook et al. 1994). The latter study states that a small quantity of geoverdin was found in a sphingid, the Verdant hawkmoth, *Euchloron megaera*, but an earlier study concluded that the water-soluble pigment in its wing scales was an anthocyanidin derivative obtained from plant food (Barbier 1984). The green pigment is located in the forewings, as the hindwings have an orange colour, due to a pigment absorbing in the UV- and blue-wavelength range (Fig. 4A). The spectra suggest that the latter pigment acts as a spectral filter in the forewings (Fig. 4A).

Carotenoids and bile pigments are quite universal among insects, as in the orthopteran Oak bush-cricket, *Meconema thalassinum* (Fig. 4B), but why is this not the case in butterflies? A possible answer to this question may be the coverage of butterfly wings with highly structured, chitinous scales. The lower lamina of the scales has a thickness of the order of 100 nm, and it then acts as a thin film reflector, often specifically reflecting strongly in the blue wavelength range (Stavenga 2014). By very small changes in thickness, the reflectance spectrum can be subtly tuned, which is generally not possible with pigments. Several *Morpho* species have perfected their blue reflections via multilayered scale ridges (Vukusic and Sambles 2003; Kinoshita 2008; Giraldo and Stavenga 2016). In many lycaenids, the scale lumen is structured as a multilayer, so becoming effective blue or green reflectors, dependent on their perforations (Wilts et al. 2009; Stavenga 2014), while the Green hairstreak, *Callophrys rubi*, has scales structured into gyroids, so acting as three dimensional photonic crystals (Michielsen et al. 2010). The scales of the diamond weevil, *Entimus imperialis*, have domains with a single-network diamond photonic crystal, together acting as green reflectors (Wilts et al. 2012b). Scaleless insects do not have those opportunities, but some species have found alternative inroads to creating a green colouration, as the Jewel beetle *Chrysochroa fulgidessima* achieves this with elytra having melanin-chitin multilayers (Stavenga et al. 2011).

The green colouration may act as a contrasting signal when surrounded by a black frame, as in several *Graphium* species, but generally it will serve for camouflage against a background of green leaves, which are green due to carotenoid combined with chlorophyll. For insects, applying an optical method similar to that of leaves is clearly the preferred way to be green (Jin and Fujiwara 2017). However, for most butterfly species, realizing a green display with the method of structural colouration may offer too attractive of a possibility to avoid.

## Data Availability

This article has no additional data.
